# HRTEM Microstructural Characterization of β-WO_3_ Thin Films Deposited by Reactive RF Magnetron Sputtering

**DOI:** 10.3390/ma10020200

**Published:** 2017-02-17

**Authors:** A. Faudoa-Arzate, A. Arteaga-Durán, R.J. Saenz-Hernández, M.E. Botello-Zubiate, P.R. Realyvazquez-Guevara, J.A. Matutes-Aquino

**Affiliations:** Física de Materiales, Centro de Investigación en Materiales Avanzados Av. Miguel de Cervantes Saavedra 120, Complejo Industrial Chihuahua, Chihuahua 31136, Mexico; alejandro.faudoa@cimav.edu.mx (A.F.-A.); alvaro.arteaga@cimav.edu.mx (A.A.-D.); joselin.saenz@cimav.edu.mx (R.J.S.-H.); eugenia.botello@cimav.edu.mx (M.E.B.-Z.); paula.realyvazquez@cimav.edu.mx (P.R.R.-G.)

**Keywords:** β-WO_3_, thin films, HRTEM, microstructural

## Abstract

Though tungsten trioxide (WO_3_) in bulk, nanosphere, and thin film samples has been extensively studied, few studies have been dedicated to the crystallographic structure of WO_3_ thin films. In this work, the evolution from amorphous WO_3_ thin films to crystalline WO_3_ thin films is discussed. WO_3_ thin films were fabricated on silicon substrates (Si/SiO_2_) by RF reactive magnetron sputtering. Once a thin film was deposited, two successive annealing treatments were made: an initial annealing at 400 °C for 6 h was followed by a second annealing at 350 °C for 1 h. Film characterization was carried out by X-ray diffraction (XRD), high-resolution electron transmission microscopy (HRTEM), scanning electron microscopy (SEM), and atomic force microscopy (AFM) techniques. The β-WO_3_ final phase grew in form of columnar crystals and its growth plane was determined by HRTEM.

## 1. Introduction

Tungsten trioxide (WO_3_), either in bulk, thin films or as nanospheres, is widely studied for applications in catalysis [1], electrochromic devices [2], and gas sensing [3], among others. For all these applications, the required physical properties are determined by the microstructure and by the present crystalline phases; however, there are very few studies on the microstructural and crystallographic characteristics of WO_3_ thin films. WO_3_ has a perovskite-type crystal structure, where some atomic displacements and rotations of the WO_6_ octahedra normally occur so that, depending on temperature, a monoclinic, triclinic, tetragonal, orthorhombic, cubic, or hexagonal symmetries can be found. Previous studies in bulk WO_3_ [4] reported the following crystalline transitions as temperature varies: monoclinic (ε-WO_3_, <−43 °C) → triclinic (δ-WO_3_, from −43 °C to 17 °C) → monoclinic (γ-WO_3_, from 17 °C to 330 °C) → orthorhombic (β-WO_3_, from 330 °C to 740 °C) → tetragonal (α-WO_3_, >740 °C).

Phase transitions between WO_3_ polymorphs have been reported to be partially reversible [5]. For WO_3_ in bulk, monoclinic (γ-WO_3_) and triclinic (δ-WO_3_) phases have been reported to be the more stable phases at room temperature. Thermal treatment of γ-WO_3_ causes a phase transformation to either orthorhombic (β-WO_3_) or tetragonal (α-WO_3_) phases. However, bulk samples do not retain any of these phases (α- and β-WO_3_) when they are cooled to room temperature. In contrast to what happens to bulk WO_3_, all of the above phases (α- and β-, γ- and δ-WO_3_) can be present in WO_3_ thin films at room temperature, depending on synthesis conditions, showing a diversity of morphologies, crystalline structures, and space groups [5].

This work is focused on the study of the morphology of a reactive RF sputtered β-WO_3_ thin film and the effects of subsequent annealing on the orthorhombic crystal structure.

## 2. Experimental

WO_3_ thin films were deposited on 5 × 7 mm Si(100)/SiO_2_ substrates using a reactive RF magnetron sputtering system (AJA, ORION 3, 13.6 MHz) with a 2-inch diameter W target (99.99% purity). The sputtering chamber was evacuated to a base pressure of 3.19 × 10^−7^ Pa. Depositions were made for 1 h with a magnetron power of 60 W. The increase of the magnetron power causes the increase of the crystallite size in the films [6]; thus, a 60 W magnetron power was chosen to obtain small crystals sizes for gas sensing. The target-to-substrate distance was fixed to 150 mm, and the substrate temperature was kept at 300 °C. A substrate temperature of 300 °C was chosen to increase the nucleation rate and so to obtain small crystals of the β-WO3 phase [7]. The sputtering atmosphere consisted of an Ar–O_2_ gas mixture with flow rates controlled by separated gas flow-meters in order to adjust the Ar–O_2_ ratio to 3:1. The total chamber pressure was fixed to 0.66 Pa. The flow rate and the total chamber pressure were chosen from a previous work, where Manno et al. [8] concluded that the best pressure is 0.66 Pa and the flow rate is 30% O_2_. 

After film depositions, two successive annealing treatments were made: an initial annealing at 400 °C for 6 h with a cooling rate of 2 °C/min (T1), followed by a second annealing at 350 °C for one 1 and cooled down up to room temperature by opening the door of a Thermolyne model 4600 furnace (T2). The purposes of annealing treatments were, first, to allow the crystallization of the thin film and, secondly, to obtain the β-WO_3_ phase. During the first annealing treatment, the sample crystallized as krasnogorite, an allotropic form of WO_3_, and a second annealing treatment was necessary to obtain the desired phase β-WO_3_. 

Samples as-deposited, the annealed at 400 °C, and the re-annealed at 350 °C were characterized by Grazing Incidence X-Ray Diffraction (GIXRD) using a PANalytical diffractometer with Cu Kα radiation; the grazing angle was set at 0.2° over an angular range from 20° to 90°, with a scan step of 0.02° and a 10 s per step counter time.

For the Rietveld refinement with the Fullprof program, the instrumental widening of the diffraction peaks was taken into account. Initial cell parameters were taken from the ICSD card #50730 for β-WO_3_ phase and from the ICSD card #836 for the krasnogorite phase. 

Surface morphology was examined and film thickness was determined by atomic force microscopy (AFM) (VEECO model SPM Multimode) and scanning electron microscopy (SEM) (JSM-7401F), respectively.

Specimens cross sections of WO_3_ films grown on Si(100)/SiO_2_ for HRTEM were prepared in a Focused Ion Bean system (JEM-9320FIB). High resolution transmission electron microscopy (HRTEM) was performed with a JEOL JEM-2200FS system.

## 3. Results and Discussion

Structural Characterization

**Figure 1:**
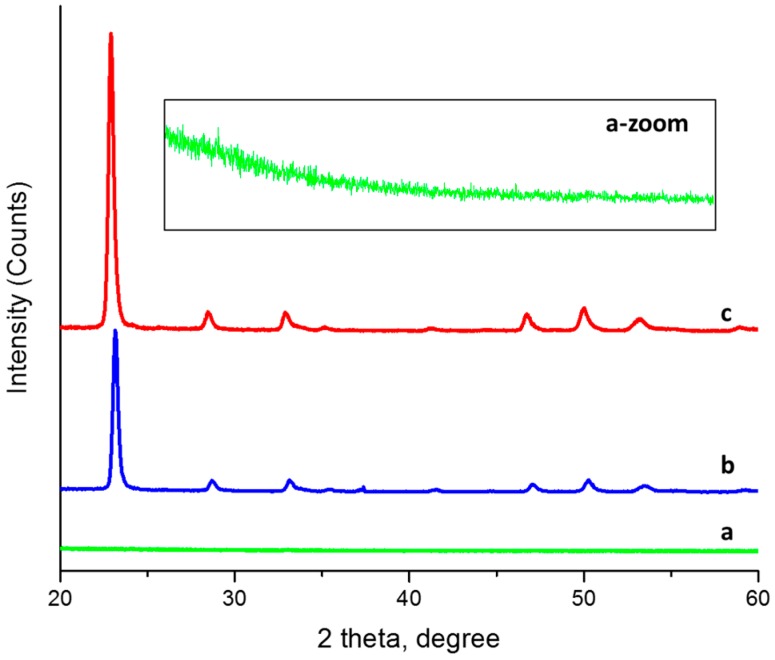
XRD of WO_3_. Thin films: (**a**) As-deposited; (**b**) First thermal treatment T1; (**c**) Two-step annealing T1 + T2. Inset a-zoom shows the zoomed DRX of the as-deposited sample.

The X-ray diffraction curves 1a and 1a-zoom show the amorphous nature of the as-deposited sample. During the annealing treatment T1, Figure 1b, the WO_3_ thin film crystallized into the krasnagorite orthorhombic phase [9], with space group *Pmnb* and unit-cell parameters *a* = 7.341 Å, *b* = 7.570 Å, and *c* = 7.754 Å. The X-ray diffraction pattern shown in Figure 1c corresponds to the WO_3_ thin film after the two successive annealing treatments, T1 and T2. The β-WO_3_ orthorhombic phase was identified with space group *Pcnb* [10] and unit-cell parameters of *a* = 7.3612, *b* = 7.5739, and *c* = 7.7620. Bulk β-WO_3_ is stable in the temperature range from 320 °C to 720 °C. However, in WO_3_ thin films, this phase can occurs in different temperature ranges [11]. 

**Figure 2:**
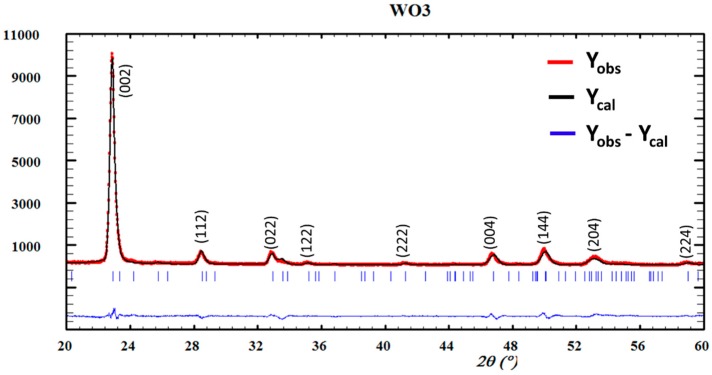
Measured and simulated XRD patterns of β-WO_3_.

Figure 2 shows the Fullprof Rietveld refinement of the GIXRD pattern of the β-WO_3_ phase. All the peaks were indexed to the β-WO_3_ phase. The sharp diffraction peaks indicate a high crystallinity degree of the sample; no impurity peaks were detected in the pattern. The strong and dominating nature of the peak corresponding to the (002) reflection indicates the preferential *c*-axis orientation of crystallites. The preferential growth of *c*-axis orientation in WO_3_ films can be understood by the lowest surface energy [7]. Additionally, the preferential growth orientation can be calculated from the texture coefficient, *TC* (*hkl*), which can be determined using the following relation [12]:

(1) $$TC\left(hkl\right)=\left(\frac{\frac{I_{\left(hkl\right)}}{I_{0\left(hkl\right)}}}{\frac{1}{N}\sum \frac{I_{\left(hkl\right)}}{I_{0\left(hkl\right)}}}\right)$$

where *I*_(*hkl*)_ is the measured relative intensity of diffraction in a plane (*hkl*), *I*_0(*hkl*)_ is the standard intensity of diffraction in the plane (*hkl*) taken from the ICSD data, and N is the reflection number. The *TC*(*hkl*) values were calculated for all diffraction planes; the (002) diffraction plane has the highest value, with *TC* = 3.06, followed by the (004) diffraction plane with *TC* = 1.51 and then by the (204) diffraction plane with *TC* = 1.13. It is clear, from the definition of the texture coefficient, that the deviation of *TC*(*hkl*) from unity implies that the film growth occurs in a certain preferred orientation. The highest TC value for the (002) diffraction plane indicates that the crystallites are preferentially oriented.

**Figure 3:**
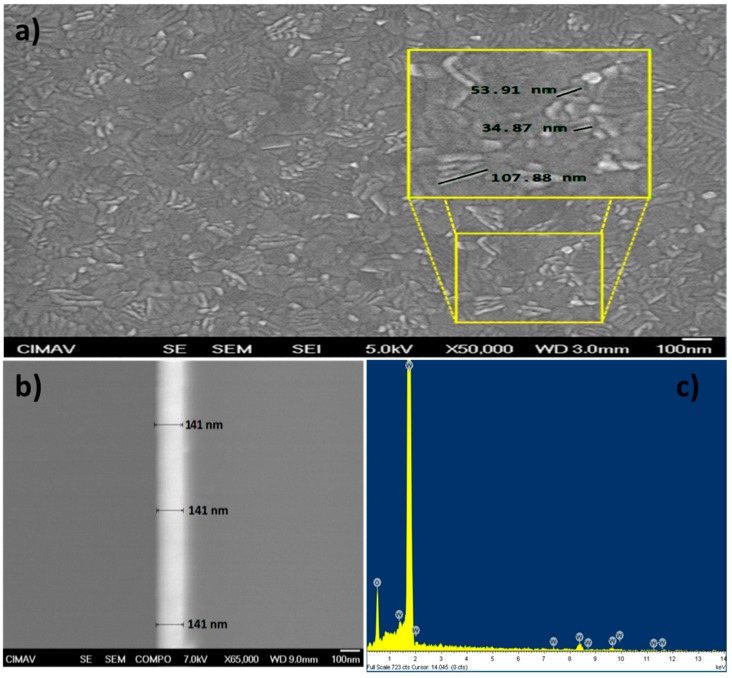
SEM image of β-WO_3_ thin film surface after the two-step annealing treatment. (**a**) Micrographs of the β-WO_3_ thin film with a zoomed area that shows the grain size; (**b**) β-WO_3_ thin film transversal area obtained with backscattered electrons; (**c**) β-WO_3_ thin film EDS spectrum.

Figure 3a shows a SEM image of the β-WO_3_ thin film surface after the two-step annealing treatment. The surface morphology is predominantly laminar, with a length distribution from 50 nm to 150 nm. Laminar structures in WO_3_ thin films are usually observed for oxygen concentrations in the sputtering chamber below 50% [13]; in this work, the oxygen concentration was 25%. A smaller amount of rounded grains with sizes below 50 nm were also observed. 

Figure 3b shows a thin film cross section taken with backscattered electrons, where the bright zone corresponds to the β-WO_3_ thin film with a film thickness of 141 nm throughout the area. This film thickness was obtained after depositing for 1 h under the above described conditions. Figure 3c is an energy-dispersive X-ray spectroscopy (EDS) elemental microanalysis, showing the characteristic W and O lines. The absence of characteristic lines of other elements shows that the film does not contain impurities. The high intensity observed for the W characteristic line is actually due to the overlap of the W Mα line with the Kα line of the Si substrate. The oxygen characteristic line originates from both the WO_3_ thin film and the small SiO_2_ layer on the Si substrate [14]. 

**Figure 4:**
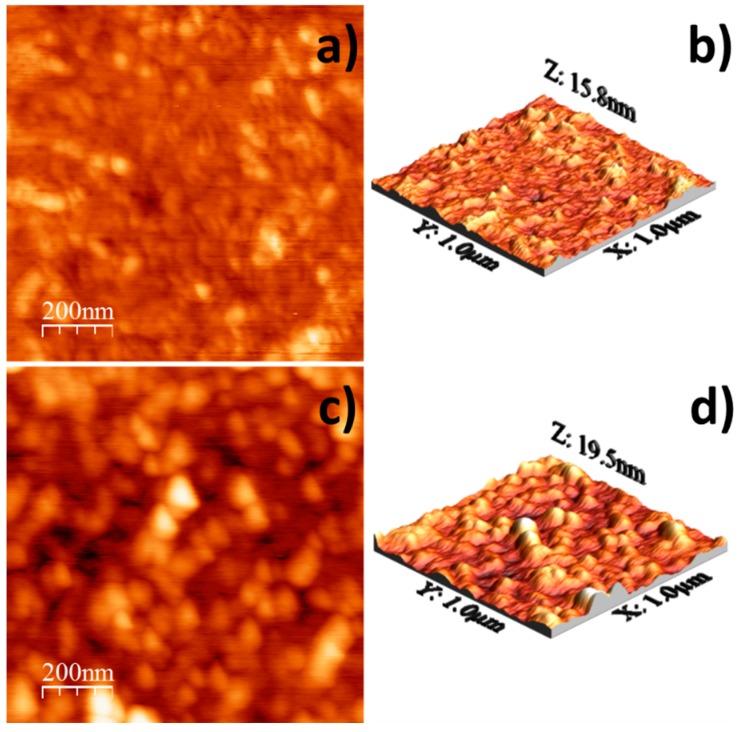
(**a**) AFM 2D of as-deposited thin film; (**b**) AFM 3D of As-deposited thin film; (**c**) AFM 2D after annealing; (**d**) AFM 3D after annealing.

Figure 4a–d show 2D and 3D AFM images (scanning area of 1 μm × 1 μm) of the as-deposited and of the two-step annealed (T1 + T2) samples. Figure 4a,b for the as-deposited film show a smooth film surface, some grains with grain boundaries that are not well defined and grain sizes in the range 40–60 nm. The root-mean-square (RMS) of the surface roughness is 1.4342 nm, with a highest roughness height of 15.8 nm.

Figure 4c,d for the two-step annealed film show a rough surface, grains sizes in the range 80–110 nm and well-defined grain boundaries. It has been previously reported that the surface roughness increases as the annealing temperature increases [15]. RMS value of the surface roughness is 2.7058 nm, and the highest roughness height is 19.5 nm. In sum, the RMS surface roughness increased from 1.4342 nm for the as-deposited film to 2.7058 nm for the two-step annealed film.

**Figure 5:**
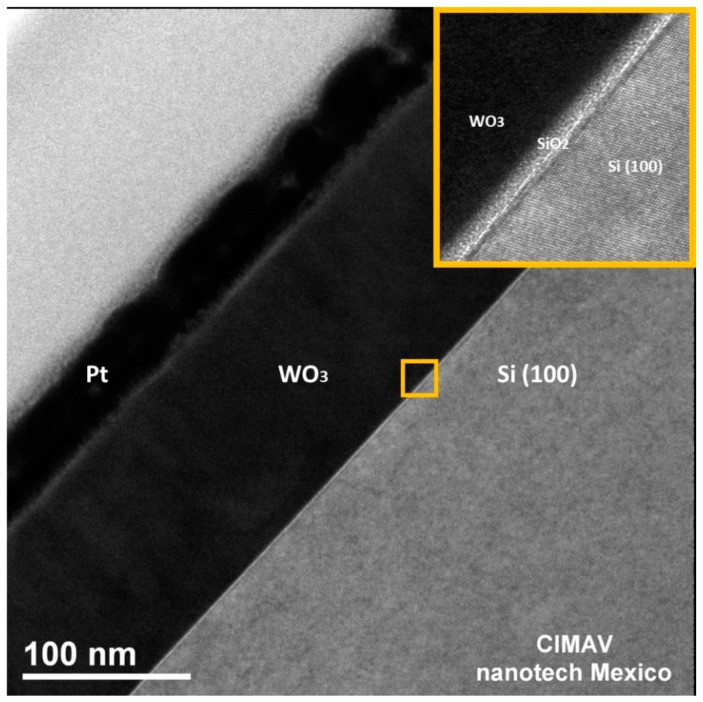
HRTEM micrograph of the cross section of the β-WO_3_ thin film.

Figure 5 shows a HRTEM image of the β-WO_3_ film cross section, where various layers are indicated. The lower right bright corner is the (100) silicon substrate. The magnified insert with the Si/WO_3_ interphase shows a 3-nm-thick amorphous layer of SiO_2_, which is originated by the oxidation of the Si surface in contact with air [16]. The next layer, above the amorphous SiO_2_ layer, corresponds to the WO_3_ thin film. Finally, the last layers are platinum/carbon protective layers deposited to protect the specimen from FIB damage.

**Figure 6:**
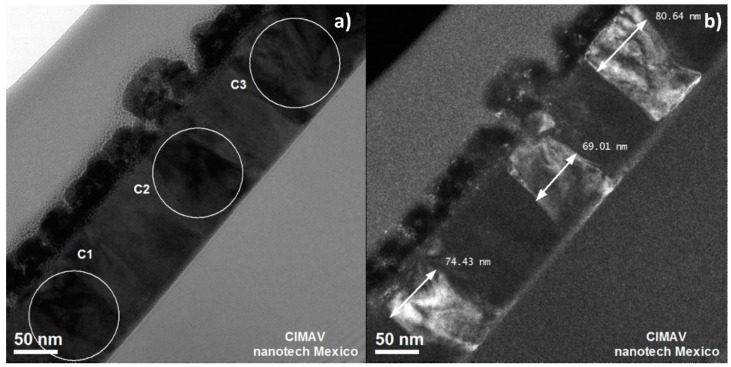
HRTEM. (**a**) Bright-field and (**b**) dark-field micrographs showing three crystals in the same orientation.

Figure 6 shows cross-section bright-field and dark-field images. In the images, three crystals, labeled as C1, C2 and C3, can be identified with roughly the same orientation. In the dark-field image, the shape and width of each crystals are particularly evident.

From the substrate, the crystals grow in columnar form. This type of columnar growth has been reported in films grown epitaxially on different crystalline substrates [17]. The β-WO_3_ film, as deposited on amorphous SiO_2_, is also amorphous, and the crystallinity appears during the annealing, as was shown above by X-ray diffraction. However, the β-WO_3_ film has a columnar growth. Thompson [18] proposed that, once the nuclei are formed, they grow into the external phase, as well as laterally in directions lying in the plane of the interface. Lateral growth leads to impingement and coalescence of crystals, resulting in the formation of grain boundaries and defining at least the initial grain-structure characteristics of the newly formed film. Usually, subsequent thickening occurs through epitaxial growth on these grains and columnar grain structures develop. Sometimes, the thickening occurs by the annealing, forming columnar grain structures, as the β-WO_3_ film. In this work, we present a detailed study about as-deposited amorphous WO_3_ on amorphous SiO_2_ substrates, and its evolution to the crystalline state during annealing, and not during deposition or with epitaxial growth. This kind of study has seldom been reported in WO_3_ crystal growth.

**Figure 7:**
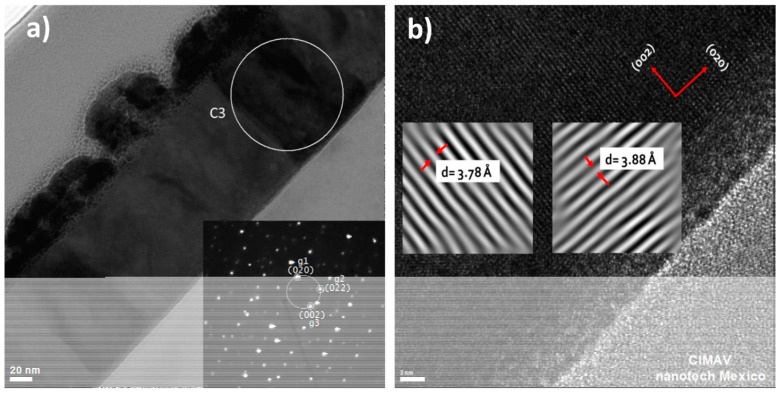
HRTEM images (**a**) with indexed diffraction pattern of crystal C3 and (**b**) showing the interplanar distance of the two planes (002) and (020).

From the electron diffraction pattern of C3-crystal, shown in Figure 7a, the crystal growth plane was determined. In fact, to define this crystal growth plane, it was necessary to identify the zone axis using the reciprocal lattice vectors (g1, g2, and g3) that represent three diffracted beams, and making their cross products. The cross product was made in the clockwise direction, because the transmitted beam is outside the circle containing the diffraction points [19]:

(2) $$\vec{B}={\vec{g}}_{1}\times {\vec{g}}_{2}.$$

According to Equation (2), the zone axis direction is [200] (in the direct beam direction that is the observation direction). Thus, by taking this observation axis perpendicular to the substrate plane, the crystal grow plane for the WO_3_ film is the (002) plane.

Figure 7b is a HRTEM image showing the (002) lattice planes parallel to the silicon substrate and the (020) lattice planes perpendicular to the substrate. The interplanar distances for these two sets of planes are better shown in the inserts after digital processing using the Digital Micrograph software. The calculated interplanar distances correspond with the reported in the crystal data sheet 01-089-4480. These results coincide with the results from X-ray diffraction, where the preferential reflection correspond to the (002) plane. The same results can be obtained using crystals C1 and C2, as all of them have the same crystallographic orientation.

## 4. Conclusions

The morphology and the crystal growth evolution of WO_3_ thin films deposited on a Si substrate were studied. A 3-nm-thick layer of amorphous SiO_2_ was observed on the Si substrate. The as-deposited WO_3_ thin film was amorphous, and after a two-step annealing treatment, the WO_3_ thin film became β-WO_3_. The film thickness was 141 nm, and the film did not contain any impurities. The crystals grew from the substrate to the surface of the film in columnar form parallel to the [001] direction. From the deviation of the X-ray texture coefficient from unity, it was concluded that the crystal grew with a (002) preferred orientation. After a two-step annealing, the as-deposited thin film had a predominantly laminar morphology with a length distribution from 50 nm to 150 nm, and a smaller amount of rounded grains with sizes below 50 nm. The root-mean-square surface roughness increased from 1.4342 nm for the as-deposited thin film to 2.7058 nm for the two-step annealed thin film. Until our knowledge, it is the first detailed study about amorphous WO_3_ thin films deposited on an amorphous SiO_2_ substrate, where the evolution to the crystalline state occurs during the annealing treatment, and not during the deposition process nor during the epitaxial growth.
